# Countercurrents: The Last Trial

**DOI:** 10.3390/curroncol28010031

**Published:** 2021-01-05

**Authors:** Steven A. Narod

**Affiliations:** 1 Women’s College Research Institute, Women’s College Hospital, Toronto, ON M5S 1B2, Canada; steven.narod@wchospital.ca; Tel.: +1-416-351-3765; Fax: +1-416-351-3767; 2 Dalla Lana School of Public Health, University of Toronto, Toronto, ON M5T 3M7, Canada

If you have been around long enough, you will have heard more than once that an important clinical question is about to be resolved by a study that is soon to be published. In some cases, the study is the last in a series and will settle the question once and for all.

In this case, the question is “Should screening mammography be initiated at age 40 or age 50?” Studies to date have left the question open. To be sure, the Canadian NBSS study had a separate component for women aged 40 to 49 and within that group, randomisation to mammography failed to produce the desired outcome [1] and as a consequence the study has been roundly criticized (faulty randomisation, old-fashioned equipment, etc.) [2]. Other studies did not over-sample young women and were underpowered for subgroup analyses. Many of the critics of the NBSS and the other studies anticipated that the claim that “younger was better” would be vindicated by a study out of the UK which randomised more than 100,000 women aged 39 to 41 either to annual mammography or to wait until age 50 to begin screening in the established UK screening program [3].

The UK AGE study aimed to answer the question if screening should start at age 40 or age 50 but has wider implications. The study sheds light on the question “Does mammography work at all?” Radiologists may object, but this is a simple matter of credence. If a study of women aged 40 to 48 showed a dramatic and statistically significant reduction in deaths from breast cancer with screening within this age group, surely this would be hard evidence that mammography works—that is, we would shift credence in favour of screening. Therefore, a negative result is also informative and should diminish our faith in the screening paradigm.

In the UK AGE trial, Stephen Duffy and colleagues compared 25-year cumulative mortality from breast cancer for women aged 40 to 48 who were invited for annual mammography and those who were not invited [3]. They counted deaths from breast cancers that occurred before age 48, the age at which they were eligible for screening through the National Health Service Screening Program. (That is, if a woman was screened and found to have breast cancer at age 35 and died of breast cancer at age 55, she was counted. If she was screened at age 35 and found to have breast cancer at age 51 and died at age 55, she was not counted). Overall, there was a small and non-significant difference in mortality (HR = 0.88; 95% CI: 0.74 to 1.03). A difference was noted in the first ten years post-randomisation (HR = 0.75; 95% CI: 0.58–0.97) but none thereafter. The net difference in mortality was about 0.06% at the 25-year mark.

The study falls short of answering the fundamental question, “What is the anticipated difference in the lifetime risk of dying of breast cancer if screening starts at age 50 or age 40?” To do this, we would need to compare the lifetime breast cancer mortality curves for the two groups: women who were invited to start at age 40 with those who were invited to start at age 50 by the NHS screening program. We would include all breast cancer deaths. These data would then allow us to directly compare the impact of a new program where screening is initiated at age 40 with the current one, where screening begins at age 50.

The study showed a slightly positive effect over the short term but this is not enough for the authors to convince us that this is supportive of early screening. They conclude that “Reducing the lower age limit for screening from age 50 to 40 years old could potentially reduce breast cancer mortality”. This is not a stellar endorsement (after all, this is the last trial).

Suppose there is a 25% reduction in the ten-year mortality of deaths from breast cancer diagnosed before age 50 with an annual mammogram. The cumulative risk of getting breast cancer between age 40 and 50, according to SEER, is 0.9% (900 per 100,000). The ten-year survival for these women is 88%; therefore, we expect 108 women in 100,000 to die of a breast cancer diagnosed in their forties. Additionally, if we prevent one quarter of these, that is 27 deaths per 100,000 women who undergo up to ten annual screens. Roughly one for every 4000 women.

I agree with Alan Hollingsworth that if breast screening works for women over age 50, it should also work for women under age 50 [2]. There may be issues of cost and false positives and negative biopsies, but whether it works is best measured by the hazard ratio (the proportion of deaths prevented) and there is no biologic reason why the hazard ratio associated with screening should be less protective for young women than for older women. After all, survival after breast cancer in young women is worse than survival for older women and the cancers in young women are often more aggressive [4]. The case against screening young women is largely based on yield and not on clinical course. If it works for women over 50, it should work for those under 50. Of course, the corollary is that if it does not work for women under 50, it does not work for women over 50.

The lead author, Stephen Duffy, has contributed widely to the mammography literature and is a long-standing champion of mammography. He has written many studies, but unfortunately the different studies do not fit together in a coherent whole. In the AGE study, the best estimate of the hazard ratio was 0.88, which is remarkably similar to that of 0.85 generated through meta-analysis by the Canadian Task Force [5]. The Task Force also concluded that the hazard ratio did not differ by age group.

In the AGE trial, all the benefits of mammography accrued to women in the first ten years since randomisation and, after that, the risk ratio was close to unity. This is in stark contrast to the Swedish two-county trial in which there was a highly significant reduction in breast cancer mortality in women invited to screening (HR = 0.69) [6]. In that study, the authors (Duffy included) concluded that the majority of the lives saved accrued ten or more years after randomisation. “*Most prevented breast cancer deaths would have occurred (in the absence of screening) after the first 10 years of follow-up… Evaluation of the full impact of screening, in particular estimates of absolute benefit and number needed to screen, requires follow-up times exceeding 20 years because the observed number of breast cancer deaths prevented increases with increasing time of follow-up”* [5].

Earlier this year in an observational study from Sweden, Duffy and his Swedish colleagues pegged the hazard ratio for mammography screening at 0.59 [7]. It would be easier for Duffy to make his case if his studies showed greater consistency.

In the past, the champions of mammography had the opportunity to stake their claim by criticizing other studies that did not show concordant results [8]. These were flawed studies, they argued, using outdated equipment, faulty methods, etc. This was an effective strategy but is hard for Duffy to invoke this strategy against his own paper.

There is a notable discordance between the results of the UK AGE trial and that of its predecessors. Until now, Stephen Duffy and Laszlo Tabar have co-authored 83 papers; to my knowledge, all have come out in favour of mammography. Moreover, nearly all positive studies for mammography come from Sweden (the exception being the early Health Insurance Plan (HIP) trial) [9]. It will be interesting to see how Laszlo Tabar reacts to the AGE trial. He can either criticize the methods or claim that a non-significant hazard ratio of 0.88 is evidence in favour of mammography.

Where do we go from here? There are no other randomised trials of screening versus no screening now underway. What are we to make of Duffy’s Last Trial? To start, Canada should opt out of mammography screening before age 50.
